# Supplementary material for: PNPLA3 is a triglyceride lipase that mobilizes polyunsaturated fatty acids to facilitate hepatic secretion of large-sized very low-density lipoprotein
Source: Nat Commun. 2024 Jun 6;15:4847. doi: 10.1038/s41467-024-49224-x (PMC11156938; doi:10.1038/s41467-024-49224-x)
Supplement: Supplementary file 1 — Supplementary Information [file 41467_2024_49224_MOESM1_ESM.pdf]

Supplementary Information

Supplementary Figure 1. PNPLA3 degrades PUFA-LDs in primary mouse hepatocytes
(a-d) Primary Hepatocytes were isolated from 10-week-old WT mice and infected with adenovirus expressing Flag-PNPLA3 WT (a-b) or I148M (c-d). Cells were treated overnight with 250 µM OA (a,c) or LA (b,d). Scale Bar =10µm. Images shown were derived from representative experiments that were repeated three times.

Supplementary Figure 2. Design and validation of PNPLA3 LKO mice. (a-b) Diagram (Biorender) of the PNPLA3 locus of PNPLA3$^{fl/fl}$ mice in the absence **(a)** or presence **(b)** of Cre recombinase. **(c-d)** PNPLA3 mRNA expression of PNPLA3$^{fl/fl}$ mice infected with AAV-TBG-null (n=6) or AAV-TBG-CRE (n=6) in brown **(c)** and white **(d)** adipose tissue. No significant difference was detected using t test.

Supplementary Figure 3. PNPLA3 knockdown does not affect liver or plasma lipid content in the absence of lipogenic stimulation. 10-week-old WT mice were fed COWD and treated with control (n=8) or PNPLA3 ASO (n=7) biweekly for 3 weeks. **(a)** Liver PNPLA3 mRNA expression. **(b)** Total liver TG content. **(c)** Total plasma TG content. ***$p<0.001$, ns= $p>0.05$, t test.

Supplementary Figure 4. Histology and gene expression of PNPLA3 knockdown livers upon LXR agonism. 10-week-old WT mice fed COWD were treated with control or PNPLA3 ASO for 3 weeks. Both groups were treated with the LXR agonist T0901317 prior to tissue collection (n=7/group). **(a-b)** Hematoxylin and eosin (H&E) staining of liver sections from Control ASO **(a)** or PNPLA3 ASO **(b)** treated mice. **(c)** Liver mRNA expression of lipogenic genes by qPCR. **(d)** Liver mRNA expression of ER stress response genes by qPCR. ***$p<0.001$, ***$p<0.0001$, t test.

Supplementary Figure 5. LD degradation by endogenous PNPLA3 in primary mouse hepatocytes requires high glucose lipogenic culture conditions. BODIPY staining of LDs in primary mouse hepatocytes treated with Control **(top)** or PNPLA3 **(bottom)**ASO. Cells were loaded with 250µM OA **(left)** or LA **(right)** in low glucose medium overnight. Scale Bar= 50µm. Images shown were derived from representative experiments that were repeated three times.

Supplementary Figure 6. Model of PNPLA3 function during lipogenesis. Lipogenic stimulation drives PNPLA3 expression, resulting in PUFA mobilization from TG for desaturation of membrane- and VLDL-PLs. PNPLA3 deficiency from knockout or I148M mutation impairs PL desaturation and VLDL lipidation, leading to hepatic retention of TG and steatosis. Schematic model was created by using Biorender.

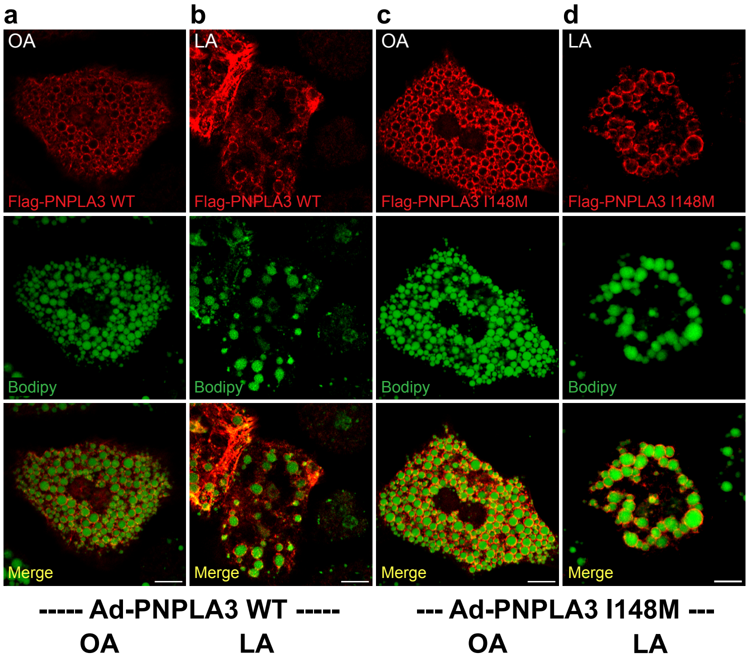

Supplementary Figure 1

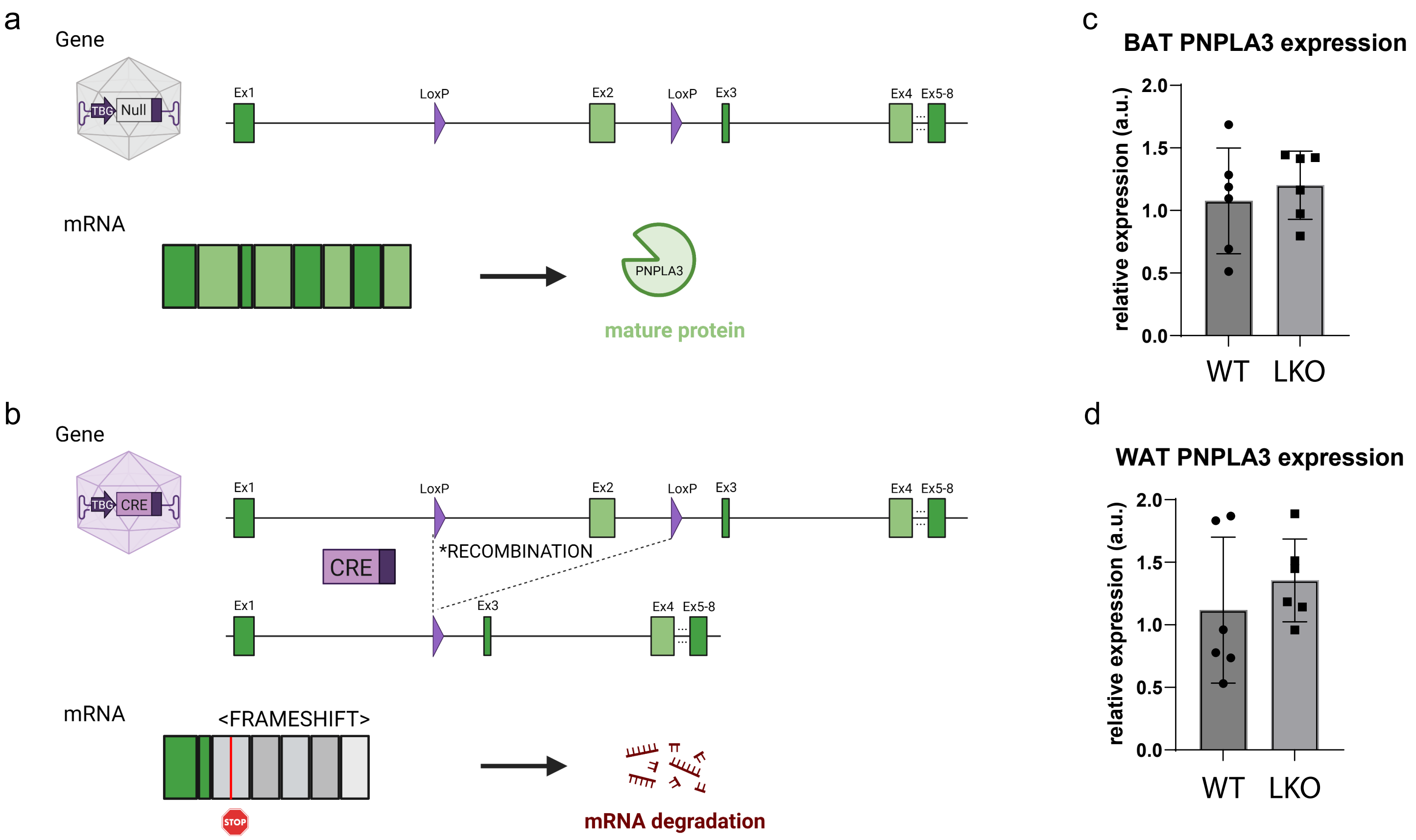

Supplementary Figure 2

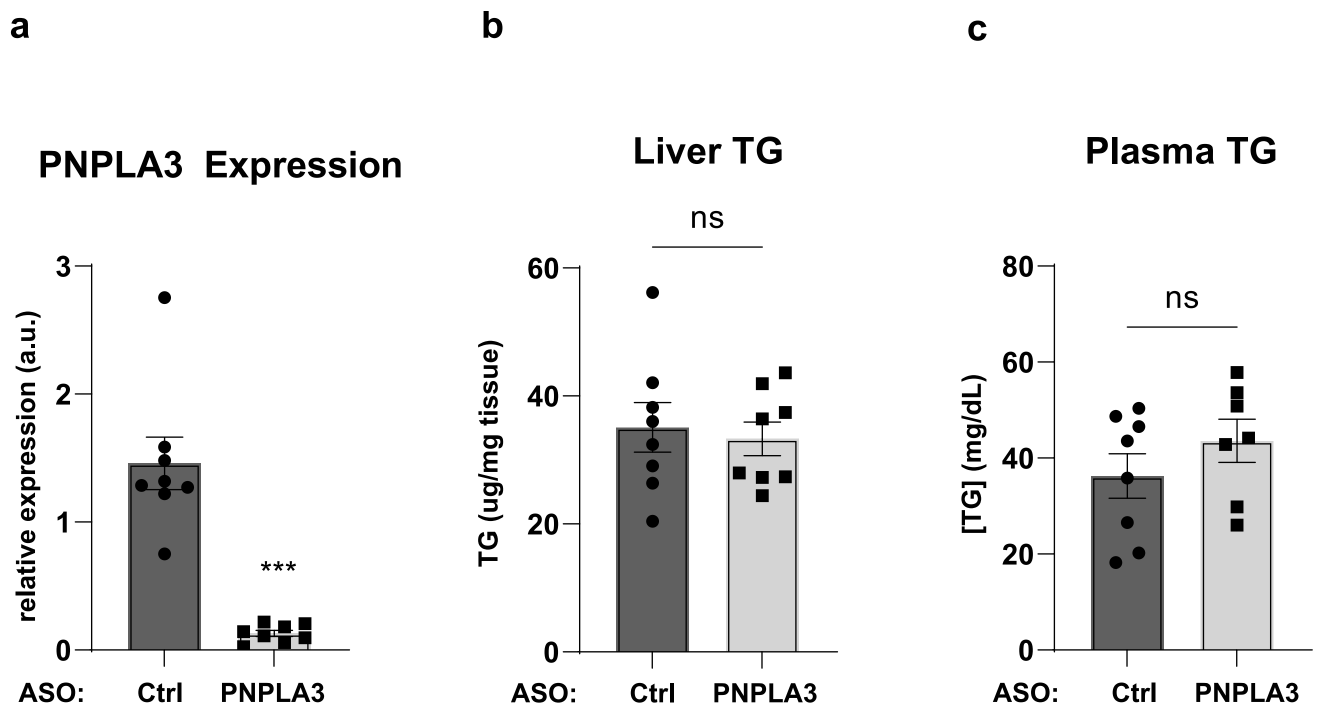

Supplementary Figure 3

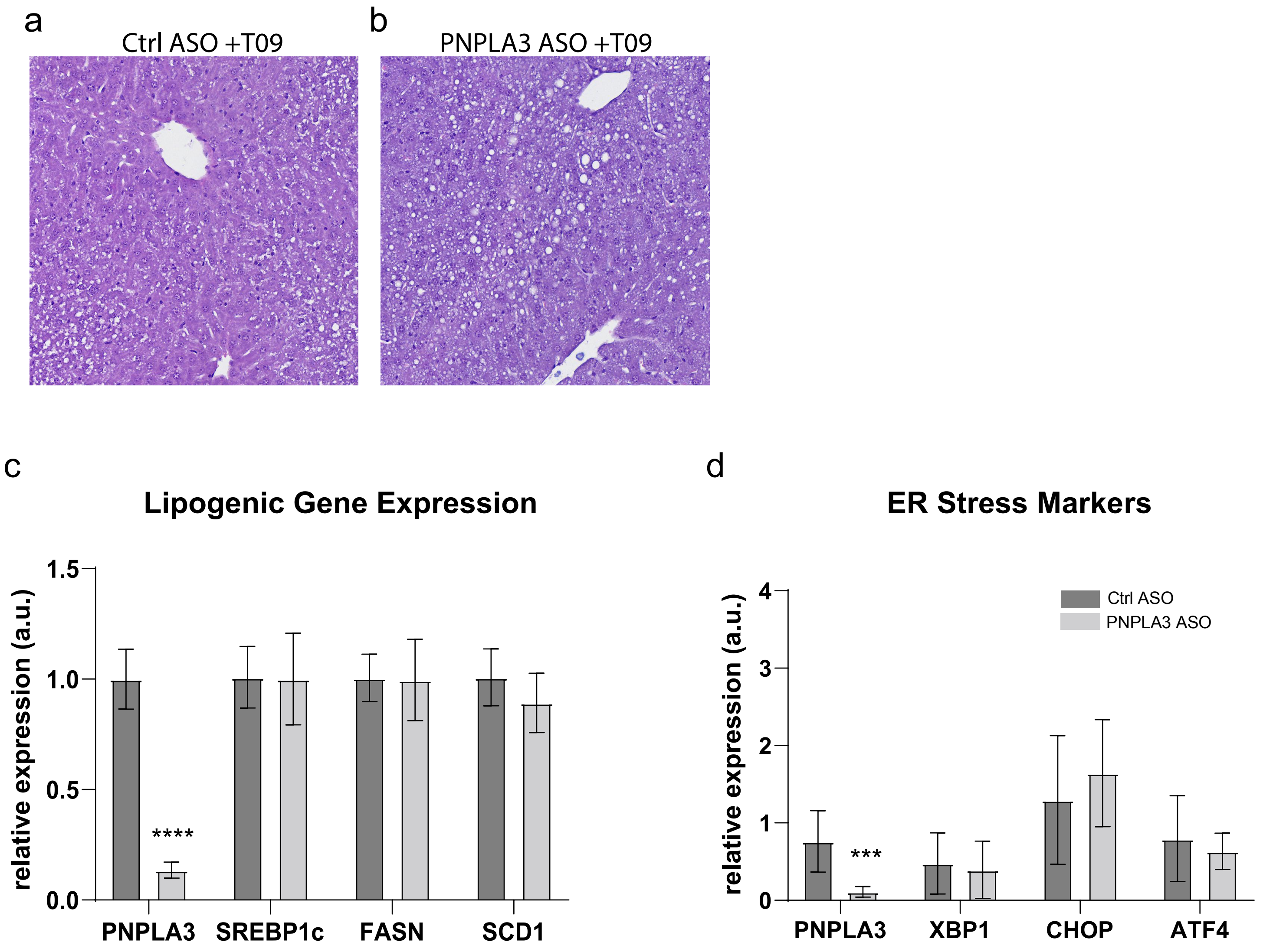

Supplementary Figure 4

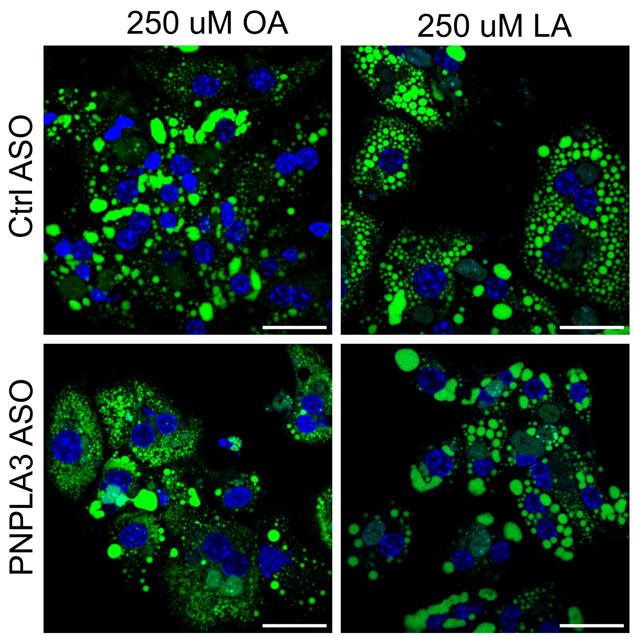

Supplementary Figure 5

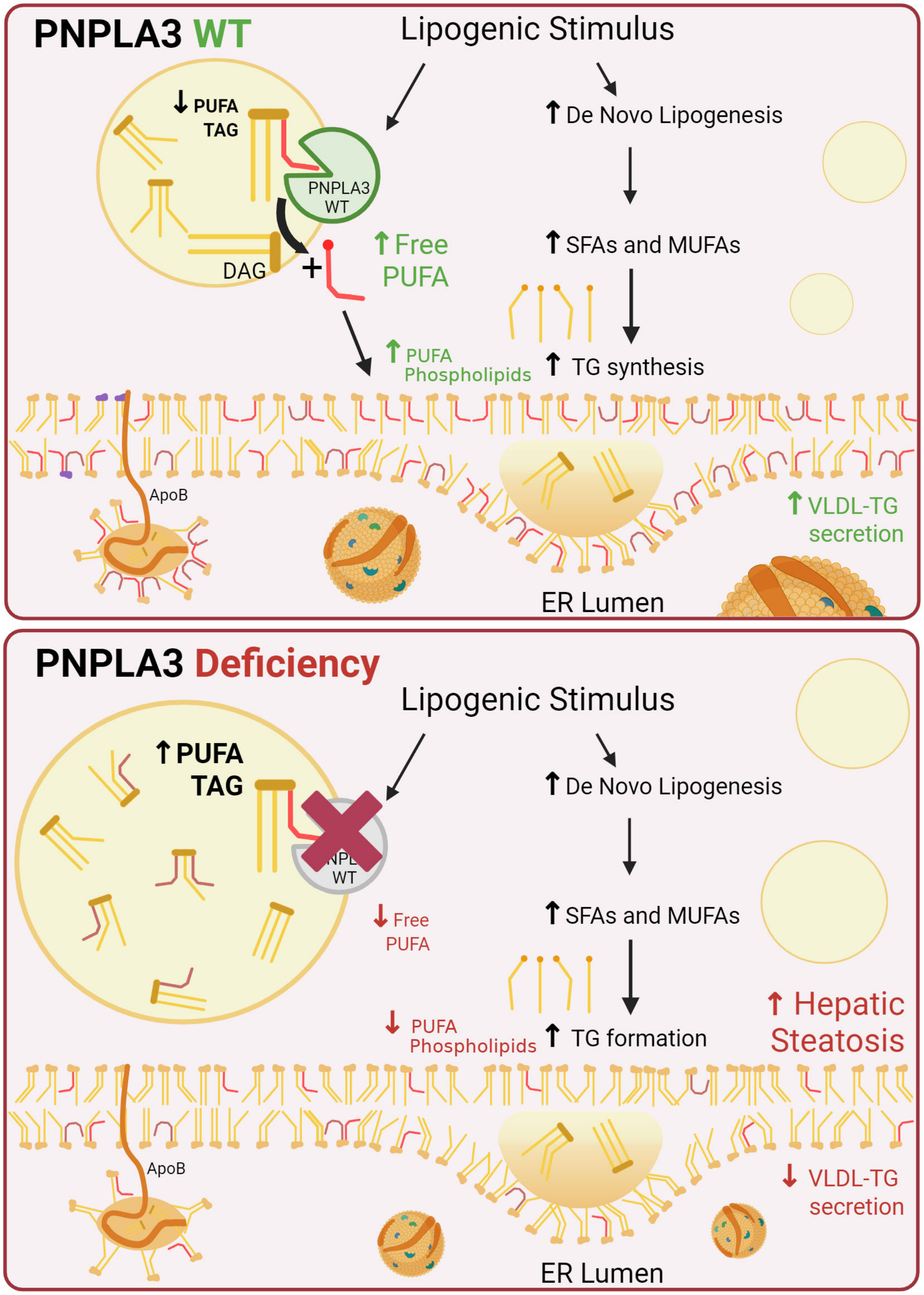

Supplementary Figure 6

Supplementary Table S1

Primer Sets for Genotyping

Primer Name	Sequence
ATGL KO_F	AGAGAGAGAAGCTGAAGCCTGG
ATGL KOwt_R	GCCAGCGAATGAGATGTTCC
ATGL KOneo_R	CTGCGTGCAATCCATCTTGT
PNPLA3fl_F	AGCTGCTAAGGTGCATTGCCA
PNPLA3fl_R	CACTCTGCCCTAGCACTTATGACAAC
PNPLA3ki_F	TACACTGGCCTGGTTCCCTTAATC
PNPLA3KI_R	GCAGAGGCAAATGTTCTGTGAG

Supplementary Table S2

Primer sets for quantitative real-time PCR

Primer Name	Sequence
PNPLA3_F	ACGTGCTGGTGTCTGAGTTCC
PNPLA3_R	AGGGACGTTGTCGCTCACTC
SREBP1_F	GCAGACCCTGGTGAGTGG
SREBP1_R	GTCGGTGGATGGGCAGTTT
ACC1_F	CTTCCTGAGAAACGAGTCTGG
ACC1_R	CTGCCGAAACATCTCTGGGA
SCD1_F	CAAGCTGGAGTACGTCTGGA
SCD1_R	CAGAGCGCTGGTCATGTAGT
FASN_F	GAGGACACTCAAGTGGCTGA
FASN_R	GTGAGGTTGCTGTCGTCTGT
ACLY_F	GCCCTGGAAGTGGAGAAGAT
ACLY_R	CCGTCCACATTCAGGATAAGA
ChREBP_F	GGCCTGGCTGGAACAGTA
ChREBP_R	CGAAGGGAATTCAGGACAGT
CHOP_F	GTCCAGCTGGGAGCTGGAAG
CHOP_R	CTGACTGGAATCTGGAGAG
ATF4_F	AGCAAAACAAGACAGCAGCC
ATF4_R	ACTCTCTTCTTCCCCCTTGC
XBP1s_F	GAGTCCGCAGCAGGTG
XBP1s_R	GTGTCAGAGTCCATGGGA
Actin_F	GGCTGTATTCCCCTCCATCG
Actin_R	CCAGTTGGTAACAATGCCATGT

Supplementary Table S3

Nutritional composition of corn-oil enriched Western diet (COWD) (D21050712i, Research Diets, New Brunswick, NJ)

Class description	Ingredients	Grams
Protein	Casein, Lactic, 30 Mesh	195.00 g
Protein	Methionine, DL	3.00 g
Carbohydrate	Sucrose, Fine Granulated	350.00 g
Carbohydrate	Lodex 10	100.00 g
Carbohydrate	Starch, Corn	50.00 g
Fiber	Solka Floc, FCC200	50.00 g
Fat	Corn Oil	210.00 g
Mineral	RD Mineral Mix (S10001A)	17.50 g
Mineral	Calcium Phosphate, Dibasic	17.50 g
Mineral	Calcium Carbonate, Light, USP	4.00 g
Vitamin	Choline Bitartrate	2.00 g
Vitamin	RD Vitamin Mix (V10001C)	1.00 g
Anti-oxidant	Ethoxyquin	0.04 g
Special	Cholesterol, NF	1.50 g
	Total:	1001.54 g
