# Supplementary material for: PNPLA3 is a triglyceride lipase that mobilizes polyunsaturated fatty acids to facilitate hepatic secretion of large-sized very low-density lipoprotein
Source: Nat Commun. 2024 Jun 6;15:4847. doi: 10.1038/s41467-024-49224-x (PMC11156938; doi:10.1038/s41467-024-49224-x)
Supplement: Supplementary file 3 — Reporting Summary [file 41467_2024_49224_MOESM3_ESM.pdf]

# nature portfolio

Corresponding author(s): Jun Liu

Last updated by author(s): Mar 12, 2024

# Reporting Summary

Nature Portfolio wishes to improve the reproducibility of the work that we publish. This form provides structure for consistency and transparency in reporting. For further information on Nature Portfolio policies, see our Editorial Policies and the Editorial Policy Checklist.

## Statistics

For all statistical analyses, confirm that the following items are present in the figure legend, table legend, main text, or Methods section.

| n/a | Confirmed | |
|---|---|---|
| ☐ | ☒ | The exact sample size (*n*) for each experimental group/condition, given as a discrete number and unit of measurement |
| ☐ | ☒ | A statement on whether measurements were taken from distinct samples or whether the same sample was measured repeatedly |
| ☐ | ☒ | The statistical test(s) used AND whether they are one- or two-sided<br>*Only common tests should be described solely by name; describe more complex techniques in the Methods section.* |
| ☒ | ☐ | A description of all covariates tested |
| ☐ | ☒ | A description of any assumptions or corrections, such as tests of normality and adjustment for multiple comparisons |
| ☐ | ☒ | A full description of the statistical parameters including central tendency (e.g. means) or other basic estimates (e.g. regression coefficient) AND variation (e.g. standard deviation) or associated estimates of uncertainty (e.g. confidence intervals) |
| ☐ | ☒ | For null hypothesis testing, the test statistic (e.g. *F*, *t*, *r*) with confidence intervals, effect sizes, degrees of freedom and *P* value noted<br>*Give P values as exact values whenever suitable.* |
| ☒ | ☐ | For Bayesian analysis, information on the choice of priors and Markov chain Monte Carlo settings |
| ☒ | ☐ | For hierarchical and complex designs, identification of the appropriate level for tests and full reporting of outcomes |
| ☒ | ☐ | Estimates of effect sizes (e.g. Cohen's *d*, Pearson's *r*), indicating how they were calculated |

*Our web collection on statistics for biologists contains articles on many of the points above.*

## Software and code

Policy information about availability of computer code

| | |
|---|---|
| Data collection | Western Blot images were captured on ImageQuatnt LAS 4000 (GE). quantitative PCR was performed on a CFX96 Real-Time System C1000 Touch Thermal Cycler(BioRad). Confocal images were taken on Zeiss LSM 980 inverted confocal microscope with Airyscan 2 (Zeiss ZEN). Colorimetric assays were detected using Epoch 96-well Microplate Spectrophotometer (BioTek) and processed using Gen5 v2.0. Radioactivity was measured using Tri-Carb 2910 TR (Perkin Elmer) and processed using QuantaSmart TM. Mass Spectrometry scans were acquired under Xcalibur software. |
| Data analysis | Statistical analysis was performed by GraphPad Prism 10. Heatmaps for lipidomics were plotted using GraphPad with values normalized to the relative abundance of each lipid species across all experimental groups to indicate relative fold change within each species. Lipid droplet and VLDL particle size was measured by ImageJ Auto Local Threshold tool (Bernsen method). Real-time PCR was analyzed by BioRad CFZ Manager v3.1. Confocal images were analyzed with ZEN (blue edition) v3.6. TEM images were processes and analyzed by SightX |

For manuscripts utilizing custom algorithms or software that are central to the research but not yet described in published literature, software must be made available to editors and reviewers. We strongly encourage code deposition in a community repository (e.g. GitHub). See the Nature Portfolio guidelines for submitting code & software for further information.

# Data

Policy information about availability of data

All manuscripts must include a data availability statement. This statement should provide the following information, where applicable:

- Accession codes, unique identifiers, or web links for publicly available datasets
- A description of any restrictions on data availability
- For clinical datasets or third party data, please ensure that the statement adheres to our policy

All data are available in the main text or the supplementary materials. Source Data are provided with this paper. Any additional information required to reanalyze the data reported in this paper is available from the lead contact upon request.

# Research involving human participants, their data, or biological material

Policy information about studies with human participants or human data. See also policy information about sex, gender (identity/presentation), and sexual orientation and race, ethnicity and racism.

| | |
|---|---|
| Reporting on sex and gender | N/A |
| Reporting on race, ethnicity, or other socially relevant groupings | N/A |
| Population characteristics | N/A |
| Recruitment | N/A |
| Ethics oversight | N/A |

Note that full information on the approval of the study protocol must also be provided in the manuscript.

# Field-specific reporting

Please select the one below that is the best fit for your research. If you are not sure, read the appropriate sections before making your selection.

☒ Life sciences ☐ Behavioural & social sciences ☐ Ecological, evolutionary & environmental sciences

For a reference copy of the document with all sections, see nature.com/documents/nr-reporting-summary-flat.pdf

# Life sciences study design

All studies must disclose on these points even when the disclosure is negative.

| | |
|---|---|
| Sample size | Sample size was included in figure legends. Sample sizes were calculated based on effect size and significance levels as assessed from prior studies and from other similar studies reported in the published literature. |
| Data exclusions | Data were excluded from analysis in instances where their PNPLA3 gene expression were clearly incongruent with expectations based on their treatment group and deviant from (outier test) the gene expression from other replicates in the same treatment group (i.e. PNPLA3 ASO did not induce gene knockdown) |
| Replication | Animal experiments were replicated using a variety of different lipogenic stimuli and genetic disruption strategies as reported throughout. Cell based studies were repeated independently at least two times for reproducibility. |
| Randomization | Mice were allocated randomly into experimental groups. for cell culture studies, each well was randomly assigned to treatment parameters in each independent round of study. |
| Blinding | In this study blinding was not possible because of the treatment of animals/cells as well as data collection were frequently done by the same investigators. Lipidomic analyses were done by collaborators and they were unaware of the study design. |

# Reporting for specific materials, systems and methods

We require information from authors about some types of materials, experimental systems and methods used in many studies. Here, indicate whether each material, system or method listed is relevant to your study. If you are not sure if a list item applies to your research, read the appropriate section before selecting a response.

## Materials & experimental systems

| n/a | Involved in the study |
|---|---|
| [ ] | [x] Antibodies |
| [ ] | [x] Eukaryotic cell lines |
| [x] | [ ] Palaeontology and archaeology |
| [ ] | [x] Animals and other organisms |
| [x] | [ ] Clinical data |
| [x] | [ ] Dual use research of concern |
| [x] | [ ] Plants |

## Methods

| n/a | Involved in the study |
|---|---|
| [x] | [ ] ChIP-seq |
| [x] | [ ] Flow cytometry |
| [x] | [ ] MRI-based neuroimaging |

# Antibodies

Antibodies used

1:1000 Rabbit anti-c-Myc, Cell Signaling, #2278 RRID:AB_490778
1:1000 Mouse anti-FLAG M2, Sigma #F1804 RRID:AB_262044
1:10000 Mouse anti-Actin, Sigma, #A1978 RRID:AB_476692
1:500 Rabbit anti-ATGL, Cell Signaling, #2138 RRID:AB_2167955
1:2000 Rabbit anti-Cre, Cell Signaling, #15036 RRID:AB_2798694
1:1000 Rabbit anti-FAS, Cell Signaling, #3180 RRID:AB_2100796
1:1000 Rabbit anti-ABHD5 (CGI-58), protientech, #12201 RRID:AB_2220710
1:1000 Mouse anti-SREBP-1 (2A4), Santa Cruz, Sc-13551 RRID:AB_628282
1:500 Mouse anti-XBP-1s, Cell Signaling, #12782 RRID:AB_2687943
1:1000 Rabbit anti-CHOP, Santa Cruz, Sc-575 RRID:AB_631365
1:1000 Goat anti-ApoB, Millipore, AB742 RRID:AB_92217
Alexa Fluor 568 donkey anti-mouse IgG (H+L), Invitrogen, A10037 RRID:AB_2534013
Alexa Fluor 488 donkey anti-mouse IgG (H+L), Invitrogen, A21202 RRID: AB_141607
Alexa Fluor 594 goat anti-rabbit IgG (H+L), Life technologies, A11037 RRID:AB_2534095
Alexa Fluor 488 goat anti-rabbit IgG (H+L), Invitrogen, A11008, RRID: AB_143165
Peroxidase AffiniPure Donkey Anti-Mouse IgG (H+L), Jackson ImmunoReserach, 715-035, 150 RRID:AB_2340770
Peroxidase AffiniPure Goat Anti-Rabbit IgG (H+L), Jackson ImmunoReserach, 111-035-003 RRID: AB_2313567

Validation

All antibodies used in this study were purchased commercially and have been validated and reported by manufacturers. Validation principles can be found on manufacturers websites, in particular at:

https://www.cellsignal.com/about-us/cst-antibody-validation-principles
https://www.sigmaaldrich.com/US/en/technical-documents/technical-article/protein-biology/elisa/antibody-standard-validation
https://www.ptglab.com/news/company-news/gen-validating-antibodies-for-specificity/
https://www.scbt.com/resources/protocols/western-immuno-blotting
https://www.emdmillipore.com/US/en/life-science-research/antibodies-assays/antibodies-overview/Antibody-Development-and-Validation/cFOb.qB.8McAAAFOb64qQvSS,nav
https://www.thermofisher.com/us/en/home/life-science/antibodies/invitrogen-antibody-validation.html

# Eukaryotic cell lines

Policy information about cell lines and Sex and Gender in Research

Cell line source(s)

Hela ATGL-/- generated in house (Zhang et al., Elife, 2017) (H. sapien,F),
Huh7 purchased from Cell Line Services (H. sapien,M)
Huh7 CGI-58-/- as a gift from Liqing Yu ((Yang et al., SciRep, 2020)(H. sapien,M)
Primary Mouse hepatocytes Isolated from male and female C57BL/6J WT, PNPLA3-LKO or PNPLA3 I148M KI mice (M. musculus, M/F)

Authentication

Knockout Cell lines were authenticated using genetic sequencing and protein knockout was confirmed using Western Blot. WT cell lines were authenticated by Cell Line Services.

Mycoplasma contamination

Cell lines were not tested for Mycoplasma contamination

Commonly misidentified lines (See ICLAC register)

No commonly misidentified cell lines were used.

## Animals and other research organisms

Policy information about studies involving animals; ARRIVE guidelines recommended for reporting animal research, and Sex and Gender in Research

| | |
|---|---|
| Laboratory animals | All mice were maintained on a 12-h light/dark cycle and given ad-libitum access to food and water as specified. All mice were metabolically synchronized by alternative 12-h fasting and 12-h refeeding for 3 days preceding tissue collection. Mice were diet challenged as indicated in text and in figure legends.<br>Mouse: C57BL/6J, Jackson Lab, Strain #:000664, RRID:IMSR_JAX:000664<br>Mouse: PNPLA3fl/fl (C57BL/6JGpt-PNPLA3em1CfloxGpt), GemPharmatech, Strain #T010917 RRID:IMSR_GPT:T010917<br>Mouse: PNPLA3-I148M-KI (C57BL/6N), Linden et al., N/A<br>Mouse: ATGL-/-, Jackson Lab, Strain #:019003 RRID:IMSR_JAX:019003<br>PNPLA3 overexpression. 10-week-old female wild-type mice (C57BL/6J)<br>PNPLA3 overexpression. 10-week-old female whole-body ATGL-/- mice (C57BL/6J)<br>PNPLA3 ASO studies. 10-week-old female wild-type mice (C57BL/6J)<br>PNPLA3-LKO studies. 8-10-week-old female PNPLA3fl/fl mice (C57BL/6J)<br>PNPLA3-I148M-KI studies. 9-12-week-old male PNPLA3-I148M-KI mice (C57BL/6N)<br>PNPLA3-I148M-KI studies. 9-12-week-old male wild-type mice (C57BL/6N) |
| Wild animals | No wild animals were used in this study |
| Reporting on sex | All experiments were conduced on female with the exception of PNPLA3 I148M knockin studies. Justification for using female mice was based on the elimination of biological sex as a variable in our experiments and on the observations that female mice were more susceptible to HSD-induced steatosis (Linden et al., Mol Metab. 2019) and that a functional interaction between ER-α and PNPLA3 p.I148M variant contributes to fatty liver disease in women (Cherubini et al., Nature Medicine 2023). We have seen that both male and female PNPLA3-LKO mice have increased liver TG and decreased plasma TG under acute LXR agonism on a COWD for 3 weeks (data not shown but available upon request), suggesting against sex-restricted phenotypes of PNPLA3 loss. On this basis, in the case of PNPLA3 knockin experiments, male mice were used due to the larger replicate number resulting from more male pups in the breeding litters and the time sensitive nature of the experiment. |
| Field-collected samples | No field-collected samples were used in this study |
| Ethics oversight | All procedures were performed according to protocols approved by the Mayo Clinic Animal Care and Use Committee (IACUC-2023-00005548). |

Note that full information on the approval of the study protocol must also be provided in the manuscript.

## Plants

| | |
|---|---|
| Seed stocks | N/A |
| Novel plant genotypes | N/A |
| Authentication | N/A |
